# Genetic instability in HSPC subpopulations of umbilical cord blood from patients with childhood acute lymphoblastic leukemia

**DOI:** 10.1038/s41598-025-88204-z

**Published:** 2025-03-15

**Authors:** Katarina Vrobelova, Lukas Jakl, Milan Skorvaga, Pavol Kosik, Matus Durdik, Eva Markova, Jana Jakubikova, Marek Holop, Miroslav Kubes, Martin Cermak, Judita Puskacova, Alexandra Kolenova, Igor Belyaev

**Affiliations:** 1 Department of Radiobiology, Cancer Research Institute, Biomedical Research Center, Slovak Academy of Sciences, Dubravska Cesta 9, 845 05 Bratislava, Slovakia; 2 Department of Tumor Immunology, Cancer Research Institute, Biomedical Research Center, Slovak Academy of Sciences, Bratislava, Slovakia; 3 Stem Cell Lab, BIOM-R, Ltd., Bratislava, Slovakia; 4 Department of Genetics, National Cancer Institute, Bratislava, Slovakia; 5 Department of Pediatric Hematology and Oncology, National Institute of Children´S Diseases and Medical Faculty, Comenius University, Bratislava, Slovakia

**Keywords:** Cancer, Cell biology, Genetics, Stem cells, Oncology

## Abstract

Preleukemic stem cells (PSC) containing preleukemic fusion genes (PFG) arise prenatally and represent the initial stage of acute lymphoblastic leukemia (ALL) development. Despite widespread efforts, the cell of origin of PFG is still unclear. For the first time, in order to identify the immunophenotype of the PSCs, different subpopulations of hematopoietic stem and progenitor cells (HSPC) of umbilical cord blood (UCB) from ALL pediatric patients and control healthy children were sorted and analyzed for the presence of diagnostically-relevant PFGs by fluorescent in situ hybridization (FISH). Representative FISH results were confirmed by RT-qPCR and validated by sequencing of the products. Not only did we identify likely subpopulations of TEL/AML1+ PSC to be CD34+ CD38+ and CD34+ CD38− cells, but we also found markedly increased instability of often associated with ALL genes in UCB HSPC subpopulations of ALL pediatric patients. Our data show that CD34+ CD38+ as well as CD34+ CD38− cells are prone to genetic instability and most likely represent the target for malignant transformation in the development of ALL. Overall, together with confirming the prenatal origin of PFGs, this study provides further insight into the preleukemic stage of ALL and shows that ALL is a potentially screen able disease.

## Introduction

Acute lymphoblastic leukemia (ALL) is one of the most common cancers in children with a peak incidence around 2 and 3 years of age^1^. The first hit in the development of ALL is most often represented by chromosomal translocation in the hematopoietic stem/progenitor cells (HSPC) still in the prenatal stage of the child’s development^2,3^. As a result, preleukemic fusion gene (PFG) is formed encoding a protein with a new or altered function that can prevent normal stem cell differentiation^3^. Leukemic transformation of PFG+ preleukemic stem cells (PSC) occurs postnatally due to secondary hits occurring only in a part of PFG carriers^4^.

The prevalence of PFG is often dependent on age of patients and can be used to classify ALL^5^. In neonates, the most common aberration connected with acute leukemias is the rearrangement of MLL gene (KTM2A)^6^. Younger children (2–5 years) often harbor t(12;21) translocation producing TEL/AML1 fusion (also known as ETV6/RUNX1) and less frequently t(9,22) leading to the formation of BCR/ABL1, widely known as Philadelphia chromosome^7^.

Despite widespread effort, the cellular origin of ALL remains unclear. Due to different prognosis as well as age-specific onset of the disease for each type of ALL, it is hypothesized that cell of origin depends on the underlying lesion^8^. Some studies have proposed the involvement of uncommitted CD34+ CD19− progenitors in ETV6/RUNX1 clone^9^, whereas others have suggested that virtually all non-B-cell progenitors in t(12,21) ALLs are ETV6/RUNX1 fusion negative^10^. For ETV6-RUNX1 however, there is also evidence that the cell of origin is CD34+ CD19+^10–12^. On the other hand, BCR-ABL fusion can arise in hematopoietic stem cells as well as B-progenitors depending on its isoform^11^. Translocations including the MLL gene, however, seem to originate earlier, in a CD34+ CD19− cells^13^. Importantly, it is generally difficult to determine the exact cell of origin for ALL subtypes. Not only the leukemic lesion can alter the cell of origin so that its properties change, but also compelling evidence comes primarily from diagnostic bone-marrow and peripheral blood analysis, which does not allow to monitor PSC before the leukemia outbreak^8^.

Characterization of PSC is important not only for understanding the onset of ALL and identifying the mechanism of disease relapse, but also for identifying the immunophenotype of PSC ultimately leading to the development of new effective therapeutic strategies^14^. Degradation of PSC should lead to a reduction in the relapse rate of the disease, since it is believed that PSC that have not been destroyed by chemotherapy, retain the proliferative potential and thus the ability to re-induce leukemic proliferation^15^. Moreover, screening of UCB and bone marrow used for transplantation for the presence of preleukemic clones might significantly reduce donor-cell derived leukemia by excluding the PFG positive UCB samples from the transplantation banks^16^. Additionally, identifying the immunophenotype of preleukemic hematopoietic stem cells (HSC) would, among other things, open the possibility to assess the risk of development of ALL in neonates.

In our previous study, mononuclear cells (MNCs) isolated from UCB of pediatric leukemic patients were tested for the presence of PFG. Using commonly used RT-qPCR method, diagnostically irrelevant PFGs were detected in UCB MNCs of patients indicating increased genetic instability of ALL associated genes as far as no such PFG were detected in UCB MNCs of matched control subjects^17^. To expand that study and identify the immunophenotype of cell of origin of PFGs, in this study we sorted UCB MNC from 4 ALL pediatric patients with and without TEL/AML1 into 8 HSPC subpopulations using FACS, expanded them on a feeder of mesenchymal stem cells and consequently analyzed for the presence of TEL/AML1, BCR/ABL and MLL rearrangements by fluorescent in situ hybridization (FISH). These steps allowed eliminating some of the limitations of our aforementioned study. Although RT-qPCR is a sensitive method, indirect analysis through the detection of fusion transcripts as well as the risk of false-positive results remains a major disadvantage. FISH successfully represents a reliable and highly specific method for direct detection of PFG. What´s more, rearrangements of genes (such as gains or deletions) were also evaluated using dual-fusion and break-apart FISH probes. In case of sufficient material, the samples were also analyzed for the presence of fusion transcript by RT-qPCR and validated by sequencing.

In summary, this study consisted of two parts: (1) the identification of TEL/AML1 + HSPC subpopulation representing the parental cell of origin of TEL/AML1 + ALL; (2) the monitoring of genetic instability in genes often associated with ALL (TEL, AML1, BCR, ABL, MLL) and their PFG. To our knowledge, this study represents the first backtrack analysis of different UCB HSPC subpopulations from pediatric ALL patients, years before the leukemia outbreak, and offers a unique insight into the origin of PFG containing PSC.

## Results

**Fig. 1:**
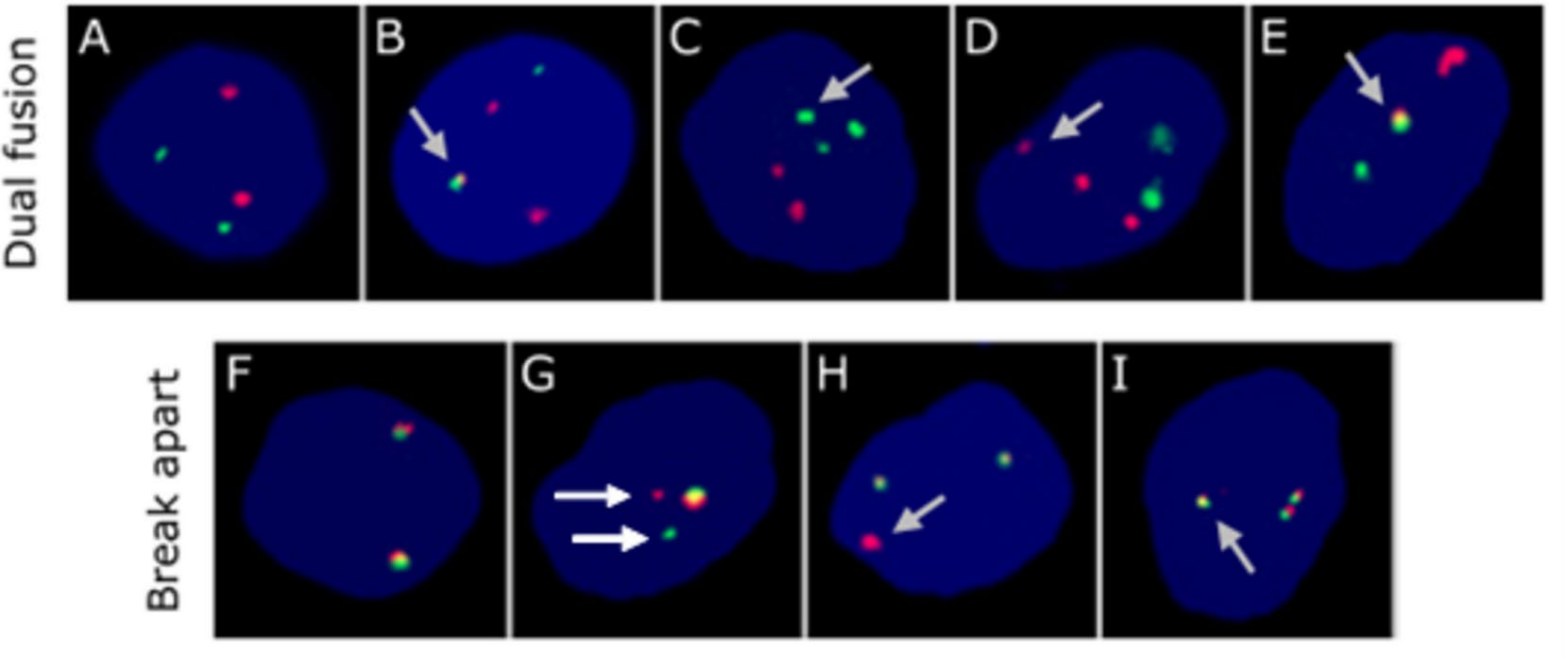
Representative images of cell nuclei analyzed with TEL/AML1 or BCR/ABL dual-fusion (**A**–**E**) and MLL break-apart (**F**–**I**) DNA. In the upper line, “dual fusion” probes labeled in red covers TEL (TEL/AML1 probe) and ABL (BCR/ABL probe), green covers AML1 (TEL/AML1 probe) and BCR (BCR/ABL probe). In the lower line, probe labeled in red covers a region telomeric to the MLL gene and green probe covers a region centromeric to the MLL gene. (**A**) Normal nuclei display two red and two green signals corresponding to two copies of analyzed genes (TEL/AML1 and BCR/ABL). (**B**) Chromosomal translocation is formed by breakage of one green and one red signal, each in two into smaller signals, followed by fusion of one small red and one small green signal into a larger yellow signal representing PFG (TEL/AML1, BCR/ABL) (marked with the arrow). Loss of one green signal points to nonreciprocal type of chromosomal translocation. (**C**) Gain of ABL (similar as gain of TEL) is detected by additional green signal (marked with the arrow). (**D**) Gain of BCR (similar as gain of AML1) or is detected by additional red signal (marked with the arrow). (**E**) An accidental overlap of one allele of TEL and AML1 (likewise BCR/ABL) (marked with an arrow) without rearrangements of these genes. (**F**) Normal nucleus two intact copies of MLL gene marked by two yellow signals, each created by overlap of one green and one red signal, (**G**) Rearrangement of MLL gene is characterized by break-down of one yellow signal, green and red fluorescent signals being visualized separately (marked with the arrow). (**H**) Gain of telomeric part of MLL gene is characterized by gain of one red signal (marked with an arrow). I: Gain of whole MLL gene is visualized as appearance of yellow signal consisting of one green and one red fluorescent signal (marked with an arrow).

HSPC (Lin-CD45+ CD34+ CD19−) subpopulations from UCB samples of 4 ALL patients and 10 healthy subjects were sorted, expanded, and analyzed for TEL/AML1, BCR/ABL PFG and MLL rearrangements by FISH. Representative images are shown on Fig. 1 A–I. In the cases of sufficient amount of material, the data were validated by RT-qPCR followed by sequencing of the resulting products.

In Patient 1 (female, 59 months at first diagnosis) TEL/AML1+ cells were detected by FISH exclusively in progenitors (1.5%) (Suppl. Tab. 1). After calculating the proportion of progenitors i the total amount of cells before sorting, TEL/AML1 positive cells accounted for 0.0029% of the MNC. In contrary, we did not observe positivity in progenitors using RT-qPCR. (Suppl. Tab 2.) Other subpopulations except for differentiated nuclear non-specify lineage negative cells (NNLNC) (Lin-CD45+ CD34-CD19−) were tested negative for TEL/AML1 by FISH (Suppl. Tab. 1) and these were also found negative for TEL/AML1 by RT-qPCR (Suppl. Tab. 2). The TEL/AML1 positivity of NNLNC was confirmed by sequencing. MNC were positive for AML1 gains (0.3%) as analyzed by FISH. The cells harboring this aberration were also detected at higher level in HSPC, Pre-Pro B cells (Lin-CD45+ CD34+ CD19+), progenitors (Lin-CD45+ CD45RA-CD34+ CD38+) and hematopoietic stem cells and multipotent progenitors (HSC/MPP) (Lin-CD45+ CD45RA-CD34+ CD38−). In addition, genetic instability was identified in NNLNC, progenitors, HSC (Lin-CD45+ CD45RA-CD34+ CD38-CD90+) and multipotent progenitors (MPP) (Lin-CD45+ CD45RA-CD34+ CD38-CD90−) using both BCR/ABL and MLL probes (Suppl. Tab. 3 and 4). Our data suggest that potential leukemogenic subpopulations in UCB of patient 1 are HSPC, Pre-Pro B cells, progenitors and HSC/MPP.

Available UCB MNC obtained from Patient 2 (male, 45 months at first diagnosis) allowed us sorting of progenitors, HSC/MPP, HSC and MPP. TEL/AML1 + cells were identified exclusively in HSC/MPP using FISH (0.1% of evaluated cells) (Suppl. Tab. 1). However, this positivity in HSC/MPP was not confirmed by validation using RT-qPCR analysis. On the other hand, RT-qPCR confirmed the negativity of the remaining subpopulations except MPP cells, where positivity was detected (Suppl. Tab. 2), but not confirmed by sequencing. MNC were tested positive for TEL gains (0.25%), which were also detected at higher level in all analyzed subpopulations except for HSC, specifically in progenitors (0.2%), HSC/MPP (0.2%) and MPP (0.4%) (Suppl. Tab. 1). Genetic instability was also analyzed by FISH using BCR/ABL (Suppl. Tab. 3) and MLL probes (Suppl. Tab. 4). Gains of BCR (Fig. 1D) and ABL (Fig. 1C) genes were found at the same level (0.3%) in progenitors (Suppl. Tab. 3). Duplications of the red part of the MLL probe (0.333%) (Fig. 1H) and whole MLL gene (0.333%) were detected in Pre-Pro B cells. In addition, cells with gains of the green part of the MLL probe were detected in progenitors (0.4%) (Suppl. Tab. 4). This data revealed subpopulations, namely progenitors and Pre-Pro B cells, with the highest genetic instability.

Lymphoblasts of the ALL Patient 3 (male, 47 months at first diagnosis) contained TEL (82.5%), AML1 (82.5%), and ABL (70%) gene duplications at the diagnosis. Low number of the Patient’s 3 UCB MNC allowed us to have sorted only B-lymphocytes, NNLNC, progenitors and HSC/MPP, although number of sorted CD34- subpopulations wasn´t sufficient for FISH analyses. MNC were tested positive for TEL/AML1 (0.25%) (Suppl. Tab. 1), while this was not confirmed by RT-qPCR. Progenitors were tested negative by FISH, due to very low prevalence. On the contrary, progenitors were tested positive for TEL/AML1 fusion using RT-qPCR and confirmed by sequencing (Suppl. Tab. 2). Sorted CD34+ subpopulations (except for progenitors) were positive for gains of TEL and AML1 genes (Suppl. Tab.1). MNC were negative for any BCR/ABL rearrangements, while progenitors were positive for BCR and ABL gene duplications (0.67%) and HSC/MPP were positive for BCR gene duplication (1%) (Suppl. Tab. 3). Notably, we found relatively high number of MLL rearrangements not only in MNC (6.67%) but also in progenitors (1.5%) and HSC/MPP (4.17%) (Suppl. Tab. 4). In this sample, the subpopulation containing the most cells with the similar genes rearrangements as blasts in diagnosis, specifically duplication of red part of the probe was identified in progenitors (2.5%) and HSC/MPP (3.33%). Also 1% of progenitor cells contained deletion of whole MLL gene.

Patient 4 (male, 120 months at first diagnosis) had no chromosomal rearrangements related to our research at the time of diagnosis. Despite MNC being negative for TEL/AML1 using FISH and RT-qPCR, TEL/AML1++ cells were identified in approximately half of the analyzed subpopulations. TEL/AML1+ cells were identified in HSPC (0.2%), Pre-Pro B cells (0.2%), HSC (0.4%), and MPP (0.2%) using FISH (Suppl. Tab. 1). The RT-qPCR results support the data obtained by FISH and sequencing revealed the presence of TEL/AML1 fusion transcript in all the analyzed subpopulations except Pre-Pro B cells (Suppl. Tab. 2). Moreover, TEL/AML1, BCR/ABL PFGs as well as MLL gains and deletions were detected in most of the analyzed subpopulations (Suppl. Tab. 1, 3 and 4). Patient 4 showed the highest degree of genetic instability of ALL associated genes in HSPC reaching 38.6% of aberrant cells in HSC/MPP, which is the most probably the leukemogenic subpopulation in this patient (Suppl. Tab. 1, 3 and 4).

While analyzing the samples we have observed cells with random overlap of either TEL and AML1 genes or BCR and ABL genes. In these cases, nuclei exhibited 3 signals, one green, one red and one yellow, with the yellow one reflecting sporadic overlapping of the red and green signal, thus suggesting spatial proximity of genes, which is not caused by chromosomal translocation (Fig. 1E). In the Patients 1–3, such cells did not exceed 2% in either of analyzed subpopulations. Since the overlapping signals were visible at the same plane, we excluded overlap caused by the same horizontal but different vertical coordinates inside the cells. Interestingly, in the Patient 4, we identified much higher number of overlapping (for TEL and AML1: 3% in Sp2, 7.4% in Sp3, 6% in Sp5, 7.6% in Sp7; and for BCR and ABL: 4.7% in Sp3, 3.8% in Sp4, 9.8% in Sp5, 9.2% in Sp6, 12.8% in Sp7, 7.6% in Sp8). It is known that individual genes have their own chromosome territories in nucleus, and the positional effect could influence the formation of fusion genes^18^. Therefore, we can assume that the likelihood of physical contact between TEL and AML1 (BCR and ABL) gene territories in a fraction of HSPC (which represents subpopulations of progenitors, HSC/MPP, HSC and MPP) of Patient 4 was increased and might contribute to the development of acute leukemia.

**Fig. 2:**
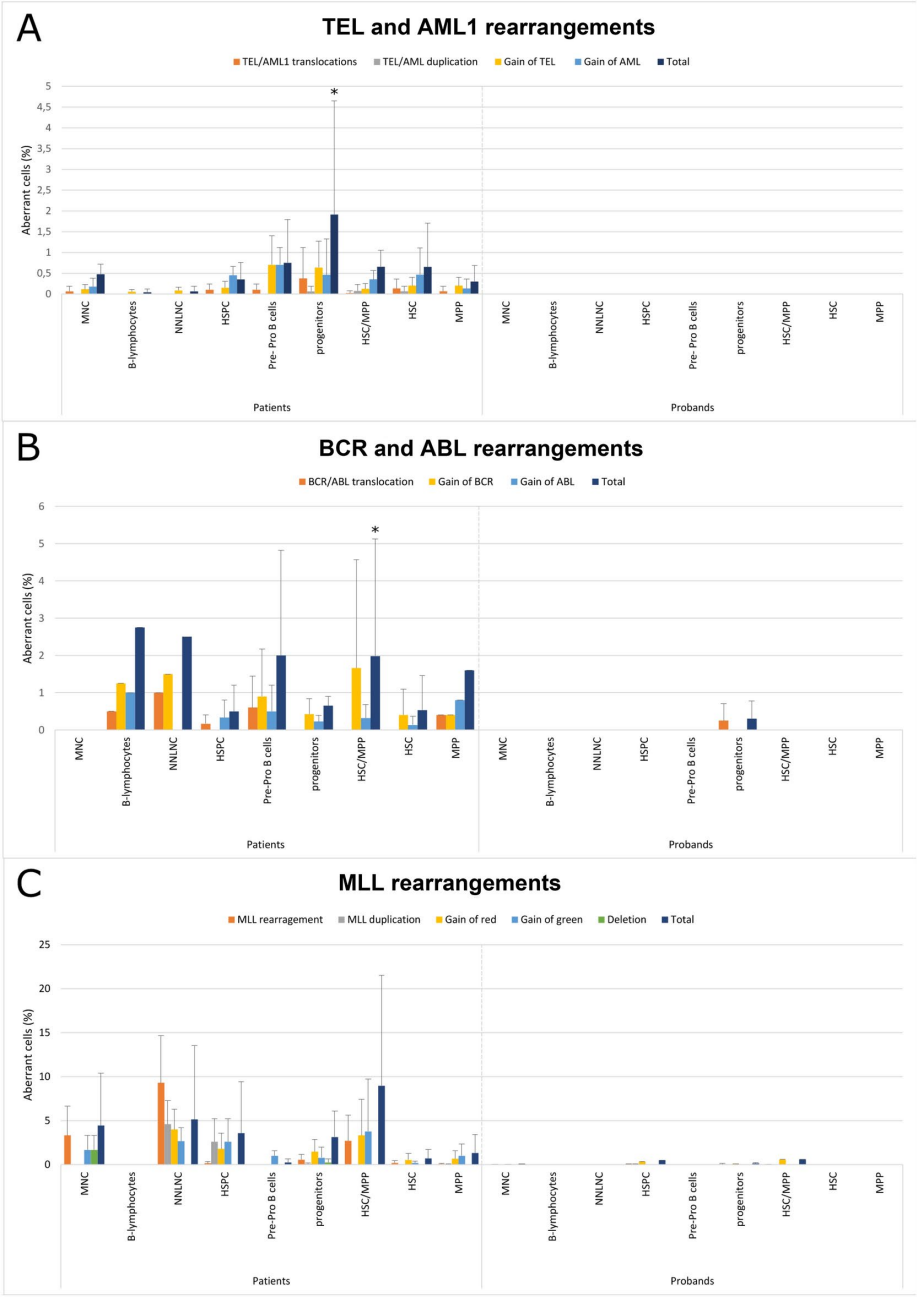
Genetic instability in different HSPC subpopulations of UCB from patients as compared to healthy subjects. Graphs show average percentages of aberrant cells for each analyzed UCB HSPC subpopulation of 4 ALL patients and 10 healthy probands. The same subpopulations were analyzed using: (**A**) TEL/AML1 dual-fusion probe, (**B**) BCR/ABL dual-fusion probe, and (**C**) MLL break-apart probe. Chromosomal translocations and gains of investigated genes were analyzed. Statistically significance (*p* < 0.05) is marked by *.

Control HSPC subpopulations from healthy subjects were screened for the presence of the same PFG. The FISH revealed a small number of sporadic genetic changes, namely rearrangements of BCR, ABL, and MLL genes (Suppl. Tab. 1, 3 and 4). These changes were identified mostly in progenitors and HSC/MPP. The RT-qPCR revealed no TEL/AML1 fusion transcripts in tested subpopulations of healthy probands. Results from ALL patients, regardless of their genetic examination at the time of diagnosis and presence of PFG, were compared to data obtained from healthy children (Fig. 2). For statistical analysis, we pooled our FISH data with the results of another three healthy probands tested for the presence of BCR-ABL by the same method in our previous study^19^.

## Discussion

Although prenatal formation of some PFG as a first hit in the development of ALL has previously been demonstrated^2,17,20–22^, the identification of cell of origin is still missing. Identifying the immunophenotype of preleukemic stem cells (PSC) is important not only for early detection of the disease, but also for extending treatment options, as PSC are likely to trigger leukemia relapse^16^.

The TEL/AML1 is the most common PFG in pediatric patients and occurs in approximately 25% B-ALL^23^. The cell of origin of TEL/AML1 is still the subject of debate, as leukemia is diagnosed postnatally and the course of its natural evolution from the cell of origin has not been followed so far. Available data about cell of origin comes primarily from studies of diagnostic bone marrow and peripheral blood studies, often with inconsistent results^8^. One hypothesis is that childhood TEL/AML1 positive B-ALL arises in CD34+ CD38− CD19+ cells associated with the early stage of B cell development^11,12,24^. This hypothesis is supported by clinical studies showing that ALL with a good prognosis, such as TEL/AML1+ ALL, develops in steroid receptor lymphoid progenitors that are prone to undergo apoptosis^10^. On the other hand, in patients with prognostically unfavorable subtypes, myeloid-like leukemias may arise in a pluripotent progenitor/stem cell resistant to apoptosis^25,26^. Our previous data show the HSC/MPP are more sensitive to ionizing radiation induced apoptosis in comparison with progenitors^27^. Other types of studies show that TEL/AML1 occurs during B-cell development before the rearrangements of IgH and TCR genes^28^. However, it has not yet been possible to distinguish between multipotent stem cells or committed progenitors as cellular target for the origin of leukemic stem cells because the expression of CD34, CD19 and CD38 may vary within HSC^29–31^. Other studies have analyzed a combination of CD markers, and the role of CD34+ CD19− progenitors has been suggested^13^. However, a possible shortcoming of such studies includes risk of subpopulation’s contamination by blasts during cell sorting. In addition to the usually used markers (CD34, CD38, CD19), we also incorporated CD90 into experiments to distinguish the stem cell subpopulations at the top of lymphocyte’s development^32^.

Importantly, identified PFG+ subpopulations do not necessarily represent the parent cell of origin being the leukemic stem cells responsible for disease relapse. Therefore, we aimed to identify the cell of origin of TEL/AML1+, BCR/ABLL and MLL/− ALL in this study.

In our previous study, backtracked analysis of the UCB MNC from pediatric ALL patients revealed presence of PFG not relevant for diagnosis and thus indicated genetic instability of ALL associated genes^17^. Nevertheless, there were some limitations such as lack of HSPC analysis as well as using RT-qPCR for detecting PFG. While RT-qPCR is widely used due to its high sensitivity, it suffers of increased risk for false positivity due to possible contamination and alternative RNA splicing. To eliminate the mentioned limitations, in this study we initially sorted UCB MNC from 4 ALL pediatric patients into different HSPC subpopulations, expanded them on a feeder from mesenchymal stem cells, and analyzed for the presence of various PFG by FISH. In Patient 1 and Patient 2 (with TEL/AML1 at time of diagnosis), we identified the probable subpopulations of TEL/AML1 origin to be progenitors with immunophenotype CD34+ CD38+ (in patient 1) and HSC/MPP with immunophenotype CD34+ CD38− (in patient 2) (Suppl. Tab. 1, 3 and 4). Our data suggests that these subpopulations represent the cellular target for development of leukemia. The difference in the immunophenotype of PSC between these two patients can be explained by the interindividual variability.

To the best of our knowledge, for the first time, we have analyzed various UCB HSPC subpopulations from pediatric ALL patients for the presence of diagnostically relevant PFG. Our second goal was to monitor the genetic instability of five most common ALL- associated genes (TEL, AML1, BCR, ABL, MLL). The FISH method using dual-fusion probes allowed direct analysis at the DNA level with high specificity and the possibility to minimize false results to the level of 0%. Our results obtained by FISH method did not correlate with results obtained by RT-qPCR. One of the possible explanations is, that PFG was present in DNA, but were not transcribed to mRNA. At the same time, the given probes make it possible to monitor not only PFG but other rearrangements of the analyzed genes and thus increase the range of identified genetic instability. In the statistical analysis, we were interested in whether patients, regardless of their genetic background, have increased genetic instability of specific HSPC subpopulation compared to healthy subjects. Significantly increased genetic instability was identified in the progenitors (*p* < 0.05) and HSC/MPP (*p* < 0.05) by the TEL/AML1 probe and BCR/ABL probe, respectively. We included 7 healthy probands in the statistical analysis, using also the results of 3 probands (together 10 healthy probands) published in our previous study, where healthy probands UCB were tested for the presence of BCR/ABL^19^. Genetic instability of BCR, ABL and MLL genes has been identified in healthy probands at a negligible level in line with the fact that PFGs can also occur in healthy individuals with a significantly lower frequency than the incidence of leukemias^33–35^. Thus, our data indicate high genetic instability of most of the HPSC subpopulations at the birth time of healthy infants who were diagnosed with ALL years later. All analyzed genes play an important role in hematopoiesis^36–41^, and their rearrangements may contribute to the development of the disease^5–7^. This is in line with a study, in which the presence of BCR/ABL was confirmed at the time of diagnosis as an additional aberration in TEL/AML1 positive B-ALL^42^ Importantly, we confirmed the presence of diagnostically relevant genes and their rearrangements (such as deletions or duplications) in different UCB HSPC subpopulations of all patients. These data confirm and extend the results of our previous study, where BCR/ABL was detected in MNC by RT-qPCR^17^. That suggest HSPC damage occurs in prenatal development, acquiring additional mutations during hematopoietic differentiation.

Altogether, our data provide further evidence that childhood ALL is a potentially screen able disease^43,44^ and increased genetic instability may be a potential biomarker for this screening, which does not require knowledge on individual diagnosis of overt leukemia such as immunophenotype, IgH gene rearrangement, or specific preleukemic gene mutations/chromosomal rearrangements. This PFG biomarkers may also be useful in exclusion of UCB from transplantation banks in order to eliminate potential donor cell derived leukemia^45,46^.

While this study is unique in both sample selection and it´s processing, it contains several limitations. The small sample size is most pronounced, but the limited availability of PFG+ umbilical cord blood bags from leukemia patients should be considered. Other disadvantages include the inability to analyze all HSPC subpopulations in all patients because even the entire umbilical cord bag was not sufficient to sort all these rare subpopulations. On the other hand, this study increased the number of analyzed HSPC subpopulations in comparison with simple cell sorting methods.

It is also important to note that by expanding the individual subpopulations, we were able to increase the probability of capturing a small number of aberrant cells as compared to MNC. In some cases, the expansion increased the number of aberrant cells up to 40-fold (Suppl. Tab. 5–7).

The first key finding is existence of leukemic markers in subpopulations of hematopoietic cells from UCB and reveal which subpopulation carry the preleukemic cells containing PFG. The second key finding of this study was that genetic instability identified by TEL/AML1, BCR/ABL dual-fusion and MLL break-apart probe was markedly increased in all analyzed HSPC subpopulations from the ALL patients (Fig. 2). We observed a statistically significant increase of total aberrations in progenitors and HSC/MPP for TEL/AML1 and BCR/ABL, respectively. Our results show that progenitors and HSC/MPP are more prone to genetic instability in genes associated with ALL than other cell subpopulations. These subpopulations probably represent the origin of ALL and could potentially serve as cellular target for immunotherapy.

In conclusion, we are the first to show a significantly increased amount of ALL-associated gene instability in most of the HSPC subpopulations in ALL pediatric patients at the time of birth, i.e. several years before the outbreak of the disease itself. At the same time, however, the interindividual instability of TEL, AML1, BCR, ABL and MLL genes in several subpopulations points not only to high variability, but also to a possible combination of damage required for disease development. Overall, we detected PFG and various rearrangements of all analyzed genes in all patients and observed considerable interindividual and subpopulation variability. No such genetic instability of ALL-associated genes was detected in the correspondent UCB HSPC subpopulations of healthy children.

## Subjects and methods

### Patients and control samples

The study was approved by the Ethical committee of Children’s hospital in Bratislava. All methods were performed in accordance with the relevant guidelines and regulations and accordance with the Declaration of Helsinki. UCB from newborns was drawn during normal birth after full term pregnancies and routinely frozen in liquid nitrogen in the Eurocord-Slovakia cord blood center. Children´s parents gave written informed consent to participate in the study. Diagnosis of leukemia was based on the French-American-British classification and flow cytometric immunophenotyping using a standard set of monoclonal antibodies according to the European Group for Immunological Characterization of Leukemia. At the time of diagnoses, samples from each patient were routinely analyzed for immunophenotype by flow cytometry and common gene rearrangements including PFG by FISH and PCR screening. In this study, samples from 4 patients (3 males/1female) and 10 control samples (6 males/4 females) were analyzed.

### Sample preparation

The UCB blood bag (500 ml) was proceeded according to validated UCB washing protocol (Standard operating procedure of Eurocord-Slovakia). Briefly, the whole cord blood was thawed in the 37 °C water bath, and then diluted with 10% solution of Dextran 40 (Merck, Darmstadt, GE) (1/2 of the UCB volume) and 5% solution of human albumin (1/2 of the original UCB volume) and centrifuged subsequently. Then the erythromass was discarded and the rest of the cell suspension was diluted with 37 °C PBS. MNC were isolated by gradient centrifugation on LSM (MP Biomedicals, Irvine, CA, USA) media as previously described^17^.

### Cell sorting

For each cell sorting we used approx. 40 million MNC. Following populations of HSPC were sorted using BD FACS Aria (BD biosciences, Franklin Lakes, NJ, USA) into separate tubes with 200 µl of complete RPMI media as we previously described^19^:

- B-lymphocytes (Sp1): Lin-CD45+ CD34-CD19+
- NNLNC (Sp2): Lin-CD45+ CD34-CD19−
- HSPC (Sp3): Lin-CD45+ CD34+ CD19−
- Pre-Pro B cells (Sp4): Lin-CD45+ CD34+ CD19+ 
- Progenitors (Sp5): Lin-CD45+ CD45RA-CD34+ CD38+ 
- HSC/MPP (Sp6): Lin-CD45+ CD45RA-CD34 + CD38−
- HSC (Sp7): Lin-CD45+ CD45RA-CD34+ CD38-CD90+ 
- MPP (Sp8): Lin-CD45+ CD45RA-CD34+ CD38-CD90−

**Fig. 3:**
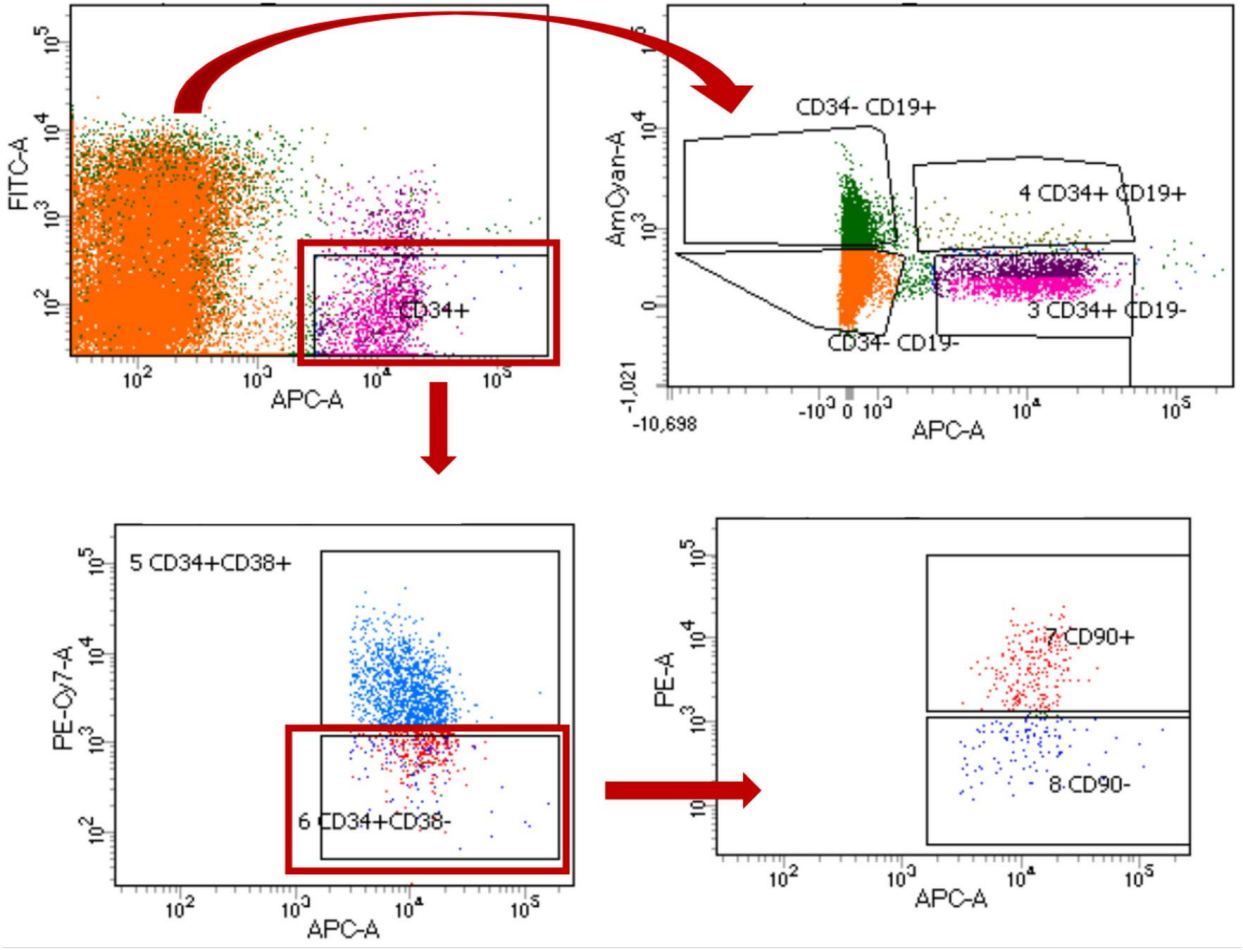
Representative image of cell sorting strategy. CD34− cells were divided into NNLNC (Sp2: CD34-CD19−) and B-lymphocytes (Sp1: CD34-CD19+). Pre-Pro-B cells (Sp4: CD34+ CD19+) and HSPC (Sp3: CD34+ CD19−) were sorted from CD45+ cells. HSPC (Sp3: CD34+ CD19−) subpopulation was subsequently divided into progenitors (Sp5: CD34+ CD38+) and HSC/MPP (Sp6: CD34+ CD38−) based on the expression of CD38, with HSC/MPP being next sorted into HSC (Sp7: CD34+ CD38-CD90+) and MPP (Sp8: CD34+ CD38-CD90−) according to CD90 expression.

The purity of cell sorting was at least 95%. In addition, 3 million of MNC were taken before sorting for analysis. Representative image of cell sorting strategy is shown in Fig. 3.

### Cell expansion

Cell expansion of rare HSPC populations was done as we previously described^19^. Briefly, the sorted cell populations were seeded on the 24-well plates in a volume of 1.5 ml of complete expansion media. Mesenchymal stem cells from umbilical cord in amount of 2 × 10^4^/well in volume of 1 ml αMEM (Merck, Darmstadt, GE) were added as “feeder” cells into each well 48 h before setting the expansion. The optimal time for harvesting was chosen before the expanded cells reach the plateau phase during the 10–12 day of expansion, when the cells contain the optimal amount of RNA.

### Fluorescent in situ hybridization

FISH was performed using directly labeled BCR/ABL, TEL/AML1 Dual Fusion probe and MLL Break Apart probe (OTG, CytoCell Aquarius, Cambridge, UK). Slide preparation was done by applying standard protocol as described previously (18). In addition to chromosomal translocations, the structure of the Dual Fusion and Break Apart probes allowed monitoring other rearrangements such as deletions, gains, and duplications of the investigated genes (Fig. 1). The slides were analyzed using fluorescent microscopy (Olympus BX51, Shinjuku, Japan) at magnification 100 × in spectrum blue, green and red. Maximum of 1000 cells were analyzed depending on the quality of the sample. Images of cells were acquired using fluorescent microscope Metafer 5.0 Systems (Metasystems, Jena, Germany) at magnification 63x.

### Fusion transcript detection

The RT-qPCR was performed as described previously^34^. However, the method of cDNA synthesis was dependent on the number of cells and thus RNA available for testing. For standard reverse transcription (RT) reaction, 1 µg of total RNA was used. In case of expanded HSPC subpopulations yielding often a limited number of cells and RNA, an amplification of cDNA has been performed for all subpopulations. Using this procedure based on Smart-seq2^47^ a controlled amplification of mRNA-derived cDNA (from 100- up to 140 000-fold) has been obtained.

### Sequencing of RT-qPCR products

Sequencing of RT-qPCR products was performed as described previously^17^ using BigDye Terminator v3.1 Cycle Sequencing Kit and manufacturer´s protocol (Applied Biosystems, Austin, TX, US). MNC and HSPC subpopulations have been considered as positive when the initial PFG positivity observed by RT-qPCR was also confirmed by sequencing of the qPCR products.

### Statistical analysis

Analysis of variance (ANOVA) with Fisher LSD were applied using Statistica software (Dell software, Round Rock, TX, US). The results were considered significantly different at *p* < 0.05.

## Supplementary Information

Supplementary Information.

## Data Availability

The datasets used and/or analyzed during the current study available from the corresponding author on reasonable request.
