# Supplementary material for: Genetic instability in HSPC subpopulations of umbilical cord blood from patients with childhood acute lymphoblastic leukemia
Source: Sci Rep. 2025 Mar 15;15:8953. doi: 10.1038/s41598-025-88204-z (PMC11910527; doi:10.1038/s41598-025-88204-z)
Supplement: Supplementary file 1 — Supplementary Information. [file 41598_2025_88204_MOESM1_ESM.pdf]

Genetic instability in HSPC subpopulations of umbilical cord blood from patients with acute lymphoblastic leukemia

Katarina Vrobelova[1+], Lukas Jakl[1+], Milan Skorvaga[1], Pavol Kosik[1], Matus Durdik[1], Eva Markova[1], Jana Jakubikova[2], Marek Holop[3], Miroslav Kubes[3], Martin Cermak[4], Judita Puskacova[5], Alexandra Kolenova[5], Igor Belyaev[1]

[1]Department of Radiobiology, Cancer Research Institute, Biomedical Research Center, Slovak Academy of Sciences, Bratislava, Slovakia

[2]Department of Tumor Immunology, Cancer Research Institute, Biomedical Research Center, Slovak Academy of Sciences, Bratislava, Slovakia

[3]Stem Cell Lab, BIOM-R, Ltd., Bratislava, Slovakia.

[4] Department of Genetics, National Cancer institute, Bratislava, Slovakia

[5]Department of Pediatric Hematology and Oncology, National Institute of Children´s Diseases and Medical Faculty, Comenius University, Bratislava, Slovakia

[+] Lukáš Jakl and Katarína Vrobelova contributed equally to this work

Supplemental table 1: Percentage of TEL andAML1 rearrangements analyzed using DNA FISH. Data show percentages of TEL/AML1 translocations, TEL andAML1 duplications, duplications of TEL segment, duplications of AML1 segment, deletions of TEL or AML1, rearrangements of TEL, rearrangements of AML1, and total **rearrangements** in each analyzed UCB HSPC subpopulation from patients and control subjects.

Sample	TEL/AML1	Subpopulation	TEL/AML1 trans. (%)	TEL/AML1 dupl. (%)	Gain of TEL (%)	Gain of AML1 (%)	Deletion TEL or AML1 (%)	Rearrangement of TEL (%)	Rearrangement of AML1 (%)	TOTAL (%)
**Patient 1**	**positive**	MNC	0	0	0	0,3	0	0	0	0,3
		Sp1	0	0	0	0	0	0	0	0
		Sp2	0	0	0	0	0	0	0	0
		Sp3	0	0	0,3	0,3	0	0	0	0,6
		Sp4	0	0	0,4	0,4	0	0	0	0,8
		Sp5	1,5	0	1,25	1,75	1,5	0	0	6
		Sp6	0	0	0	0,25	0	0	0	0,25
		Sp7	0	0	0	0	0	0	0	0
		Sp8	0	0	0	0	0	0	0	0
**Patient 2**	**positive**	MNC	0	0	0,25	0	0	0,5	0	0,75

		Sp5	0	0	0,2	0,1	0	0	0	0,3
		Sp6	0,1	0	0,2	0,2	0	0	0	0,5
		Sp7	0	0,2	0	0,2	0	0	0	0,4
		Sp8	0	0	0,4	0,4	0	0	0	0,8
**Patient 3**	**negative**	MNC	0,25	0	0	0	0	0	0	0,25
		Sp1	0	0	0	0	0	0	0	0
		Sp2	0	0	0	0	0	0	0	0
		Sp5	0	0,25	0,5	0	0	0	0	0,75
		Sp6	0	0,3	0,3	0,3	0	0	0,3	1,2
**Patient 4**	**negative**	MNC	0	0	0,2	0,4	0	0	0	0,6
		Sp1	0	0	0,167	0	0	0	0	0,167
		Sp2	0	0	0,25	0	0	0	0	0,25
		Sp3	0,2	0	0	0,6	0	0	0	0,8
		Sp4	0,2	0	1	1	0	0	0	2,2
		Sp5	0	0	0,6	0	0	0	0	0,6
		Sp6	0	0	0	0,667	0	0	0	0,667
		Sp7	0,4	0	0,6	1,2	0	0	0	2,2
		Sp8	0,2	0	0,2	0	0	0	0	0,4
**Control 1**	**negative**	Sp1	0	0	0	0	0	0	0	0
		Sp2	0	0	0	0	0	0	0	0
		Sp3	0	0	0	0	0	0	0	0
		Sp5	0	0	0	0	0	0	0	0
		Sp6	0	0	0	0	0	0	0	0
		Sp7	0	0	0	0	0	0	0	0
		Sp8	0	0	0	0	0	0	0	0
**Control 2**	**negative**	Sp1	0	0	0	0	0	0	0	0
		Sp2	0	0	0	0	0	0	0	0
		Sp5	0	0	0	0	0	0	0	0
		Sp6	0	0	0	0	0	0	0	0
		Sp7	0	0	0	0	0	0	0	0
		Sp8	0	0	0	0	0	0	0	0
**Control 3**	**negative**	MNC	0	0	0	0	0	0	0	0
		Sp1	0	0	0	0	0	0	0	0
		Sp5	0	0	0	0	0	0	0	0
		Sp6	0	0	0	0	0	0	0	0
**Control 4**	**negative**	MNC	0	0	0	0	0	0	0	0
		Sp1	0	0	0	0	0	0	0	0
		Sp2	0	0	0	0	0	0	0	0
		Sp5	0	0	0	0	0	0	0	0
		Sp6	0	0	0	0	0	0	0	0
		Sp7	0	0	0	0	0	0	0	0
		Sp8	0	0	0	0	0	0	0	0

Supplemental table 2: **RT qPCR analysis.** Table shows concentration of isolated total RNA, amount of RNA per cell, results of RT-qPCR and standard PCR for patients (P1-4) and healthy controls (K4-7) and finally the validation of PFG positivity performed by sequencing of qPCR product subcloned into sequencing vector.

	**RNA**	**RNA**		**TEL-AML1**	
	[ng/µl]	[pg/cell]	RT-qPCR	standard PCR	sequencing
**Patient1**					
MNC	82.7	0.92	-	-	-
#1	9.4[A]	0.75	0/3	-	-
#2	3.4[A]	0.09	3/3	3/3	2/3
#3	5.2[A]	0.78	0/3	-	-
#4$_{F}$	151.3	4.5	0/3	-	-

#4	100.0	3.46	0/3	-	-
#5$_F$	170.7	6.16	0/3	-	-
#5	121.6	5.86	0/3	-	-
#6	136.8	3.83	0/3	-	-
#7	122.1	3.7	0/3	-	-
#8	163.9	4.41	0/3	-	-
**P2**					
MNC	85.7	1.29	-	-	-
#2	1.6 [A]	0.05	0/3	-	-
#5	351.6	5.5	0/3	-	-
#6	240.9	3.93	0/3	-	-
#7	8.6 [lost]	0.99	-	-	-
#8	2.7 [A]	0.57	1/3	0/1	-
**P3**					
MNC	12.5	0.47	0/3	-	-
#5	97.5	3.7	2/3	2/2	2/2
#6	9.5 [A]	1.35	0/3	-	-
**P4**					
MNC	274.1	3.04	0/3	-	-
#2	12.2 [A]	0.45	1/3	1/1	1/1
#3	109.8	2.4	1/3	1/1	1/1
#4	45.5	3.5	1/3	1/1	0/1
#5	159.7	4.2	0/3	-	-
#6	208.4	8.6	0/3	-	-
#7	149.9	9.7	0/3	2/3	2/2
#8	138.6	7.2	0/3	1/3	1/1

**Control 2**	**RNA**	**RNA**	**TEL-AML1**			**MLL$_2$-AF4**			**Bcr-Abl p190**		
	[ng/µl]	[pg/cell]	qPCR	stPCR	sequencing	qPCR	stPCR	sequencing	qPCR	stPCR	sequencing
MNC	142.7	4.2	0/3	-	-	1/3	0/1	-	0/3	-	-
#5	443.3	5.5	0/3	-	-	-	-	-	-	-	-
#6	259.7	5.9	0/3	-	-	-	-	-	-	-	-
#7	80.4	5.2	0/3	-	-	-	-	-	-	-	-
#8	85.0	4.6	0/3	-	-	-	-	-	-	-	-
**Control 3**											
MNC	144.6	1.44	0/3	-	-	0/3	-	-	1/3	0/1	-
#5	237.1	5.0	0/3	-	-	-	-	-	-	-	-
#6	229.6	5.9	0/3	-	-	-	-	-	-	-	-
**Control 4**											
MNC	277.7	2.77	0/3	-	-	0/3	-	-	0/3	-	-
#5	13.6[A]	1.10	0/3	-	-	-	-	-	-	-	-

#5[lys]	8.7 [A]	1.00	0/3	-	-	-	-	-	-	-	-
#6	14.4 [A]	0.99	0/3	-	-	-	-	-	-	-	-
#6[lys]	27.1 [A]	0.57	0/3	-	-	-	-	-	-	-	-
**Control 5**											
MNC	85.8	0.86	0/3	-	-	0/3	-	-	0/3	-	-
#5	34.4 [A]	1.3	3/3	0/3	-	-	-	-	-	-	-
#6	18.3 [A]	1.1	1/3	1/1	0/1	-	-	-	-	-	-
#7	141.3	3.4	0/3	-	-	-	-	-	-	-	-
#7	141.3[A]		3/3	1/3	0/1	-	-	-	-	-	-
#7	247.6	3.2	0/3	-	-	0/3	-	-	0/3	-	-
#7	247.6 [A]		0/3	-	-	-	-	-	-	-	-

[A] cDNA was amplified (usually due to low RNA concentration); [lys] lysed erythrocytes in the sample; stPCR, standard PCR - used for amplification of qPCR product (for subcloning into a sequencing vector).

Supplemental table 3: Percentage of BCR and ABL rearrangements analyzed using DNA FISH. Table shows the percentages of BCR/ABL translocations, BCR and ABL duplications, duplications of BCR segment, duplications of ABL segment, deletions of BCR or ABL, rearrangements of BCR, rearrangements of ABL and total rearrangements in each analyzed UCB MNC subpopulation for patients and control subjects.

**Sample**	**BCR/ABL**	**Subpopulation**	**BCR/ABL trans. (%)**	**BCR/ABL dupl. (%)**	**Gain of BCR (%)**	**Gain of ABL (%)**	**Deletion BCR or ABL (%)**	**Rearrangement of BCR (%)**	**Rearrangement of ABL(%)**	**TOTAL (%)**
**Patient 1**	**negative**	MNC	0	0	0	0	0	0	0	0
		Sp2	1	0	1,5	0	0	0	0	2,5
		Sp3	0	0	0	0	0	0	0	0
		Sp4	0	0	0	0	0	0	0	0
		Sp5	0	0	0	0,4	0	0	0	0,4
		Sp6	0	0	0	0	0	0	0	0
		Sp7	0	0	0	0	0	0	0	0
		Sp8	0,4	0	0,4	0,8	0	0	0	1,6
**Patient 2**	**negative**	Sp5	0	0	0,3	0,3	0	0	0	0,6
		Sp6	0	0	0	0	0	0	0	0
		Sp7	0	0	0	0	0	0	0	0
**Patient 3**	**negative**	MNC	0	0	0	0	0	0	0	0
		Sp5	0	0	1	0	0	0	0	1
		Sp6	0	0	0,667	0,667	0	0	0	1,334
**Patient 4**	**negative**	Sp1	0,5	0	1,25	1	0	0	0	2,75
		Sp3	0,333	0	0	0,667	0	0	0	1
		Sp4	1,2	0	1,8	1	0	0	0	4
		Sp5	0	0	0,4	0,2	0	0	0	0,6
		Sp6	0	0	6	0,6	0	0	0	6,6
		Sp7	0	0	1,2	0,4	0	0	0	1,6
		Sp8	0,4	0	0,4	0,8	0	0	0	1,6
**Control 3**	**negative**	MNC	0	0	0	0	0	0	0	0
		Sp1	0	0	0	0	0	0	0	0
		Sp2	0	0	0	0	0	0	0	0
**Control 5**	**negative**	Sp3	0	0	0	0	0	0	0	0
		Sp5	1,5	0	0	0	0	0	0	1,5
		Sp6	0	0	0	0	0	0	0	0

		Sp7	0	0	0	0	0	0	0	0
		Sp8	0	0	0	0	0	0	0	0
**Control 6**	**negative**	Sp1	0	0	0	0	0	0	0	0
		Sp2	0	0	0	0	0	0	0	0
		Sp3	0	0	0	0	0	0	0	0
		Sp4	0	0	0	0	0	0	0	0
		Sp5	0	0	0	0	0	0	0	0
		Sp6	0	0	0	0	0	0	0	0
		Sp7	0	0	0	0	0	0	0	0
		Sp8	0	0	0	0	0	0	0	0
**Control 7**	**negative**	Sp3	0	0	0	0	0	0	0	0
		Sp5	0	0	0	0	0	0	0	0
		Sp6	0	0	0	0	0	0	0	0
		Sp7	0	0	0	0	0	0	0	0
		Sp8	0	0	0	0	0	0	0	0

Supplemental table 4: Percentage of MLL rearrangements analyzed using DNA FISH. Table shows the percentages of MLL rearrangements, MLL duplications, gains of red signal, gains of green signal, deletions of MLL,and total rearrangements in each analyzed UCB HSPC subpopulation of patients and control subjects.

**Sample**	**MLL**	**Subpopulation**	**MLL rearragement (%)**	**MLL dupl. (%)**	**Gain of red (%)**	**Gain of green (%)**	**Deletion (%)**	**TOTAL (%)**
**Patient 1**	**negative**	Sp1	0	0	0	0	0	0
		Sp3	0	0	0	0	0	0
		Sp5	0	0	0,1	0,1	0	0,2
		Sp6	0	0	0	0	0	0
		Sp7	0	0	0	0,2	0	0,2
		Sp8	0	0,1	0	0	0	0,1
**Patient 2**	**negative**	MNC	0	0	0	0	0	0
		Sp4	0	0,333	0,333	0	0	0,666
		Sp5	0	0	0	0,4	0	0,4
		Sp6	0	0	0	0	0	0
		Sp7	0	0	0	0	0	0
**Patient 3**	**negative**	MNC	6,667	0	0	3,333	3,333	13,333
		Sp5	1,5	0	2,5	0	1	5
		Sp6	4,167	0	3.333	0	0	4,167
**Patient 4**	**negative**	Sp3	0,333	5,2	3,6	5,2	0	14,333
		Sp4	0	0	0	1	0	1
		Sp5	0,667	0	3	3	0	6,667
		Sp6	6,667	0	10	14,667	0	31,334
		Sp7	0,6	0	1,6	0,4	0	2,6
		Sp8	0,2	0	2	3	0	5,2
**Control 8**	**negative**	MNC	0	0	0	0	0	0
		Sp1	0	0	0	0	0	0
		Sp2	0	0	0	0	0	0
		Sp5	0	0	0	0	0	0
		Sp6	0,125	0	0	0	0	0,125
		Sp7	0	0	0	0	0	0
		Sp8	0	0	0	0	0	0
**Control 9**	**negative**	MNC	0	0	0	0	0	0
		Sp1	0	0	0	0	0	0
		Sp2	0	0	0	0	0	0
		Sp3	0	0	0	0	0	0
		Sp5	0	0	0	0	0	0
		Sp6	0	0	0	0	0	0
		Sp7	0	0	0	0	0	0
**Control 10**	**negative**	MNC	0	0	0	0	0	0
		Sp1	0	0	0	0	0	0
		Sp2	0	0	0	0	0	0
		Sp3	0	0	0	0	0	0
		Sp5	0	0	0	0	0	0
		Sp6	0	0	0	0	0	0
		Sp7	0	0	0	0	0	0

		Sp8	0	0	0	0	0	0

Supplemental table 5: **Change in the number of TEL and AML1 aberrant cells before and after expansion.** Data show the results for all aberrant subpopulations for each patient. Table shows the time of expansion, derived doubling time, number of cells before and after the expansion, number of positive cells, derived total positive cells in expanded samples and in the samples before expansion.

PATIENT SAMPLE INFO			SORTING AND EXPANSION					PFG ANALYSIS FISH			ABERRATIONS ANALYSIS FISH		
**Patient**	**Sorted subp.**	**Number of cells after overnight incubation and filtration**	**Number of cells before expansion**	**Number of cells after expansion)**	**Time of expansion (h)**	**Doubling time (h)**	**Number of cell cycles**	**Relative PFG positive cells after expansion**	**Absolute PFG positive cells after expansion**	**Absolute PFG positive cells before expansion**	**Relative aberrant positive cells after expansion**	**Absolute aberrant positive cells after expansion**	**Absolute aberrant positive cells before expansion**
**Patient 1**	3	57,5 x 10^6	1218	400000	312	37,32	8,36	0	0	**0**	0,006	2400	**7,31**
	4	57,5 x 10^6	8300	1500000	312	41,61	7,50	0	0	**0**	0,008	12000	**66,4**
	5	57,5 x 10^6	11697	1330000	312	45,69	6,83	0,015	19950	**175,46**	0,06	79800	**701,82**
	6	57,5 x 10^6	2225	1870000	312	32,12	9,72	0	0	**0**	0,0025	4675	**5,56**
**Patient 2**	5	20 x 10^6	5600	240000	288	53,12	5,42	0	0	**0**	0,003	720	**16,8**
	6	20 x 10^6	1500	2320000	288	27,18	10,59	0,001	2320	**1,5**	0,005	11600	**7,5**
	7	20 x 10^6	1794	460000	288	35,99	8,00	0	0	**0**	0,004	1840	**7,18**
	8	20 x 10^6	791	240000	288	34,93	8,25	0	0	**0**	0,008	1920	**6,33**
**Patient 3**	5	5 x 10^6	1647	990000	312	33,80	9,23	0	0	**0**	0,0075	7425	**12,35**
	6	5 x 10^6	476	360000	312	32,63	9,56	0	0	**0**	0,012	4320	**5,71**
**Patient 4**	3	45 x 10^6	10310	1580000	312	42,98	7,26	0,002	3160	**20,62**	0,008	12640	**82,48**
	4	45 x 10^6	1159	580000	312	34,79	8,97	0,002	1160	**2,32**	0,022	12760	**25,50**
	5	45 x 10^6	10000	1430000	312	43,576202	7,1598713	0	0	**0**	0,006	8580	**60**
	6	45 x 10^6	4528	920000	312	40,695921	7,6666161	0	0	**0**	0,00667	6136,4	**30,20176**
	7	45 x 10^6	2092	660000	312	37,583844	8,3014394	0,004	2640	**8,368**	0,022	14520	**46,024**
	8	45 x 10^6	4035	770000	312	41,181889	7,576146	0,002	1540	**8,07**	0,004	3080	**16,14**

Supplemental table 6: **Change in the number of BCR and ABL aberrantcells before and after expansion.** Data show the results for all aberrant subpopulations for each patient. Table shows the time of expansion, derived doubling time, number of cells before and after the expansion, relative positive cells, derived total positive cells in expanded samples and in the samples before expansion.

PATIENT SAMPLE INFO			SORTING AND EXPANSION					PFG ANALYSIS FISH			ABERRATIONS ANALYSIS FISH		
**Patient**	**Sorted subp.**	**Number of cells after overnight incubation and filtration**	**Number of cells before expansion**	**Number of cells after expansion**	**Time of expansion (h)**	**Doubling time (h)**	**Number of cycles**	**Relative PFG positive cells after expansion**	**Absolute PFG positive cells after expansion**	**Absolute PFG positive cells before expansion**	**Relative aberrant positive cells after expansion**	**Absolute aberrant positive cells after expansion**	**Absolute aberrant positive cells before expansion**
**Patient 1**	5	57,5 x 10^6	11697	1330000	312	45,69	6,83	0	0	**0**	0,004	5320	**46,79**
	8	57,5 x 10^6	3266	1980000	312	33,75	9,24	0,004	7920	**13,06**	0,016	31680	**52,26**

**Patient 2**	5	20 x 10^6	5600	240000	288	53,12	5,42	0	0	**0**	0,006	1440	**33,6**
**Patient 3**	5	5 x 10^6	1647	990 000	312	33,80	9,23	0	0	**0**	0,01	9900	**16,47**
	6	5 x 10^6	476	360 000	312	32,63	9,56	0	0	**0**	0,01334	4802,4	**6,35**
**Patient 4**	3	45 x 10^6	10310	1580000	312	42,98	7,26	0,00333	5261,4	**34,33**	0,01	15800	**103,1**
	4	45 x 10^6	1159	580000	312	34,79	8,97	0,012	6960	**13,91**	0,04	23200	**46,36**
	5	45 x 10^6	10 000	1430000	312	43,58	7,16	0	0	**0**	0,006	8580	**60**
	6	45 x 10^6	4528	920 000	312	40,70	7,67	0	0	**0**	0,066	60720	**298,85**
	7	45 x 10^6	2092	660 000	312	37,58	8,30	0	0	**0**	0,016	10560	**33,47**
	8	45 x 10^6	4035	770 000	312	41,18	7,58	0,004	3080	**16,14**	0,016	12320	**64,56**

Supplemental table 7: **Change in the number of cells aberrant for MLL rearrangements before and after expansion.** Data show the results for all the aberrant subpopulations for each patient. Table shows the time of expansion, derived doubling time, number of cells before and after the expansion, relative positive cells, derived total positive cells in expanded samples and in the samples before expansion.

PATIENT SAMPLE INFO			SORTING AND EXPANSION					PFG ANALYSIS FISH			ABERRATIONS ANALYSIS FISH		
**Patient**	**Sorted subp.**	**Number of cells after overnight incubation and filtration**	**Number of cells before expansion**	**Number of cells after expansion**	**Time of expansion (h)**	**Doubling time (h)**	**Number of cycles**	**Relative PFG positive cells after expansion**	**Absolute PFG positive cells after expansion**	**Absolute PFG positive cells before expansion**	**Relative aberrant positive cells after expansion**	**Absolute aberrant positive cells after expansion**	**Absolute aberrant positive cells before expansion**
**Patient 1**	5	57,5 x 10^6	11697	1330000	312	45,69	6,83	0	0	**0**	0,002	2660	**23,39**
	7	57,5 x 10^6	804	1 720 000	312	28,20	11,06	0	0	**0**	0,002	3440	**1,61**
	8	57,5 x 10^6	3266	1 980 000	312	33,75	9,24	0	0	**0**	0,001	1980	**3,27**
**Patient 2**	4	20 x 10^6	5600	240 000	288	53,12	5,42	0	0	**0**	0,007	1680	**39,20**
	5	20 x 10^6	1500	2 320 000	288	27,18	10,59	0	0	**0**	0,004	9280	**6**
**Patient 3**	5	5 x 10^6	1647	990 000	312	33,80	9,23	0,02	14850	**24,71**	0,05	49500	**82,35**
	6	5 x 10^6	476	360 000	312	32,63	9,56	0,04	15001,2	**19,83**	0,04	15001,2	**19,83**
**Patient 4**	3	45 x 10^6	10310	1580000	312	42,98	7,26	0,01	5261,4	**34,33**	0,14	226461,4	**1477,73**
	4	45 x 10^6	1159	580000	312	34,79	8,97	0	0	**0**	0,01	5800	**11,59**
	5	45 x 10^6	10 000	1430000	312	43,58	7,16	0,01	9538,1	**66,7**	0,07	95338,1	**666,7**
	6	45 x 10^6	4528	920 000	312	40,70	7,67	0,07	61364	**302,02**	0,31	288272,8	**1418,80**
	7	45 x 10^6	2092	660 000	312	37,58	8,30	0,01	3960	**12,55**	0,03	17160	**54,39**
	8	45 x 10^6	4035	770 000	312	41,18	7,58	0,01	1540	**8,07**	0,05	40040	**209,82**
